# Use of an Implantable Loop Recorder in Facilitating the Differential Diagnosis of Isolated Cardiac Sarcoidosis: A Case Report

**DOI:** 10.7759/cureus.80444

**Published:** 2025-03-11

**Authors:** Kosuke Katano, Yuriko Sato, Taisuke Mizumura, Satoru Arai, Yoichi Sugimura

**Affiliations:** 1 Cardiovascular Center, Kawakita General Hospital, Tokyo, JPN

**Keywords:** cardiac sarcoidosis, case report, differential diagnosis, implantable loop recorder, paroxysmal atrioventricular block

## Abstract

Cardiac sarcoidosis is a relatively rare disease that can lead to arrhythmia or sudden death. Herein, we report a case of isolated cardiac sarcoidosis presenting as syncope with a paroxysmal atrioventricular block in a 34-year-old man. After reviewing the results of standard cardiac testing, we administered an implantable loop recorder, which confirmed paroxysmal atrioventricular block. Based on the information gathered from the loop recorder and subsequent testing, the patient underwent pacemaker implantation. After implantation, the ventricular thresholds rapidly increased due to the spread of inflammation related to cardiac sarcoidosis. Steroid therapy was initiated and subsequently weaned per protocol. An implantable loop recorder is commonly used to investigate the cause of unknown syncope. This modality can facilitate the identification of both the cause and the underlying disease, allowing for relatively early intervention. This case highlights the usefulness of implantable loop recorders in diagnosing or confirming the causes of syncope of unknown origin suspected to be of a cardiogenic nature.

## Introduction

Sarcoidosis is a systemic granulomatous disease of an unknown etiology. Sarcoidosis involving the heart, that is, cardiac sarcoidosis, significantly affects the prognosis of patients, as it may lead to life-threatening arrhythmias or severe heart failure and sudden death. The prevalence of cardiac sarcoidosis is higher in Japan than in Western countries. Immunosuppressive therapies using corticosteroids can delay the progression of cardiac involvement; therefore, accurate and early diagnosis is essential [1].

In Japan, cardiac sarcoidosis is more common in middle-aged and older women, which indicates clear differences between the sexes. However, no such peak in the incidence of cardiac sarcoidosis at these ages has been reported in Japanese men. Physicians should be aware that cardiac sarcoidosis is relatively common among young men in Japan [2].

The clinical manifestations of cardiac sarcoidosis include heart failure, bundle branch block, atrioventricular (AV) block, and ventricular arrhythmia, which may lead to sudden death. When the early inflammation of cardiac sarcoidosis has not yet manifested as severe clinical symptoms, the patients can present with syncope. In the early stages of cardiac sarcoidosis, determining the causes of syncope may be challenging because findings of general physical examinations are often normal. Diagnosing syncope can be very difficult, and reportedly 47% of patients with syncope remain undiagnosed even after standard cardiac testing [3]; therefore, implantable loop recorders (ILRs) are increasingly used to investigate potential causes. These devices are implanted under the skin and can detect paroxysmal arrhythmia, which is otherwise difficult to detect using standard electrocardiography (ECG) or a long-term ECG recording [4]. This report presents the case of a patient with cardiac sarcoidosis presenting with syncope. This case holds particular significance in Japan, where the incidence of cardiac sarcoidosis is relatively higher than in other countries, underscoring the importance of effective and appropriate diagnostic strategies for prompt management.

## Case presentation

A 34-year-old man with a history of malignant lymphoma in remission presented with syncope. Six months prior to presentation, he had experienced sudden loss of consciousness while running. Syncope recurred approximately once monthly regardless of the activity level - rest or exertion. Diagnostic examinations performed at other hospitals did not reveal the cause of the syncope; therefore, the patient was referred to our syncope center.

**Figure 1:**
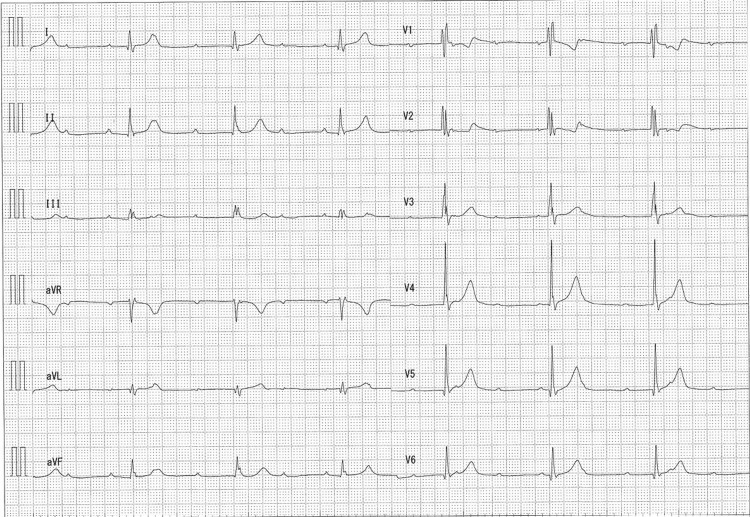
ECG at first medical examination ECG shows normal sinus rhythm with a heart rate of 92 beats per min and complete right bundle branch block. ECG, electrocardiogram

Upon admission, the patient’s vital signs included a blood pressure of 88/68 mmHg and a pulse rate of 105 beats per min. Laboratory testing revealed only mild hepatic dysfunction. ECG showed sinus rhythm with a complete right bundle branch block, and the QRS width was 124 ms (Figure 1). Moreover, the patient showed mild hypokinesis with an ejection fraction (EF) of 48%. The head-up tilt test revealed vasodepressor reflex syncope. The patient reported that this symptom was not the same as the syncope while running. He felt dizziness for a very short time; however, his syncope of natural origin was sudden in onset, and thus there were not any prodromal symptoms. Then we suspected other causes of syncope. Based on the results of these diagnostic measures, an ILR procedure was planned to evaluate the possibility of cardiogenic syncope.

**Figure 2:**
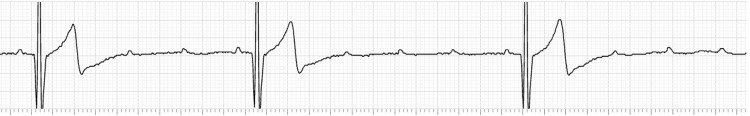
ILR findings at the time of syncope According to the ILR, QRS waves, which normally follow the P wave, were lacking; therefore, paroxysmal AV block was diagnosed. AV, atrioventricular; ILR, implantable loop recorder

One month after implantation, the ILR confirmed a paroxysmal AV block lasting for several seconds. Simultaneously, the patient lost consciousness (Figure 2). Based on the unusual occurrence of an AV block in a young man, syncope during exertion, a reduced EF on ECG, and a head-up tilt test that was negative for bradycardia, latent cardiomyopathy was suspected. Cardiac magnetic resonance imaging (MRI) was performed, and the late-enhanced image showed extensive late gadolinium enhancement, particularly in the lateral wall.

**Figure 3:**
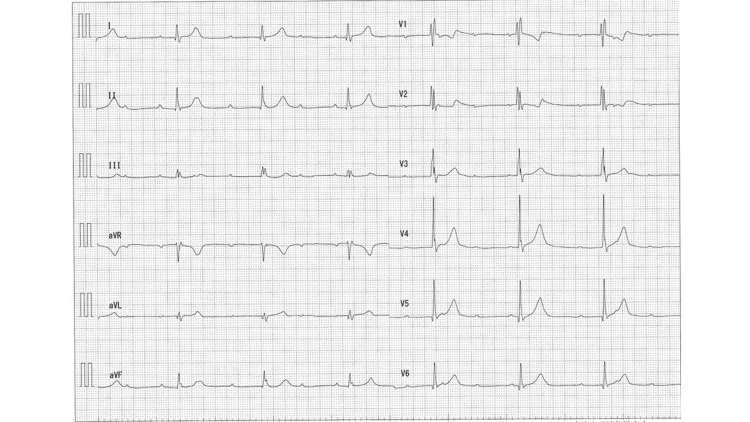
ECG two weeks after ILR implantation ECG shows a complete AV block with a heart rate of 30–40 beats per minute. AV, atrioventricular; ECG, electrocardiogram; ILR, implantable loop recorder

**Figure 4:**
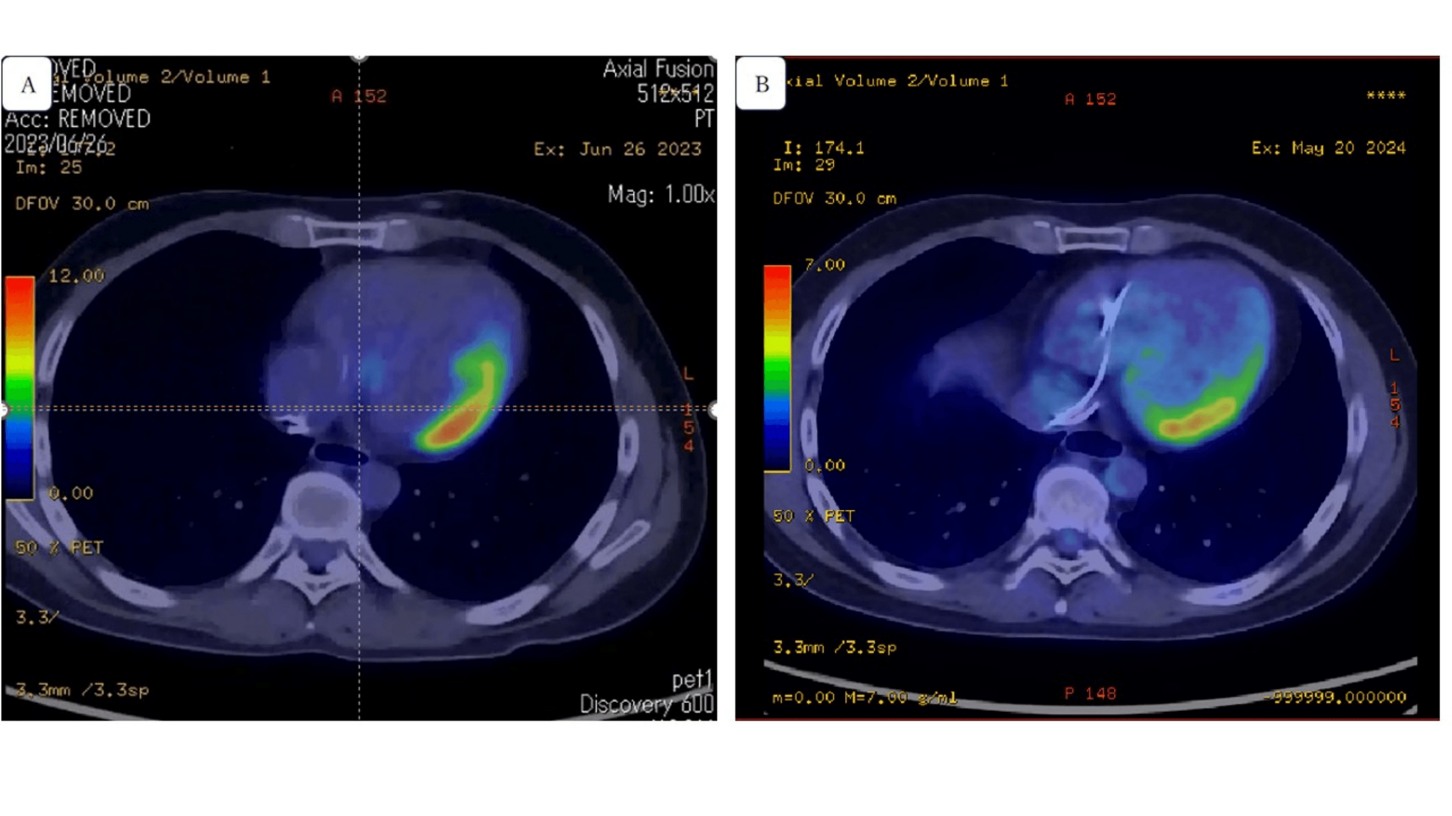
FDG-PET/CT images (A) The images show high uptake in the lateral wall (SUV max = 11.13). (B) Ten months later, follow-up images show less uptake in the lateral wall (SUV max = 5.14). FDG-PET/CT, fluorodeoxyglucose-18-positron emission tomography/computed tomography; SUV, standardized uptake value

**Figure 5:**
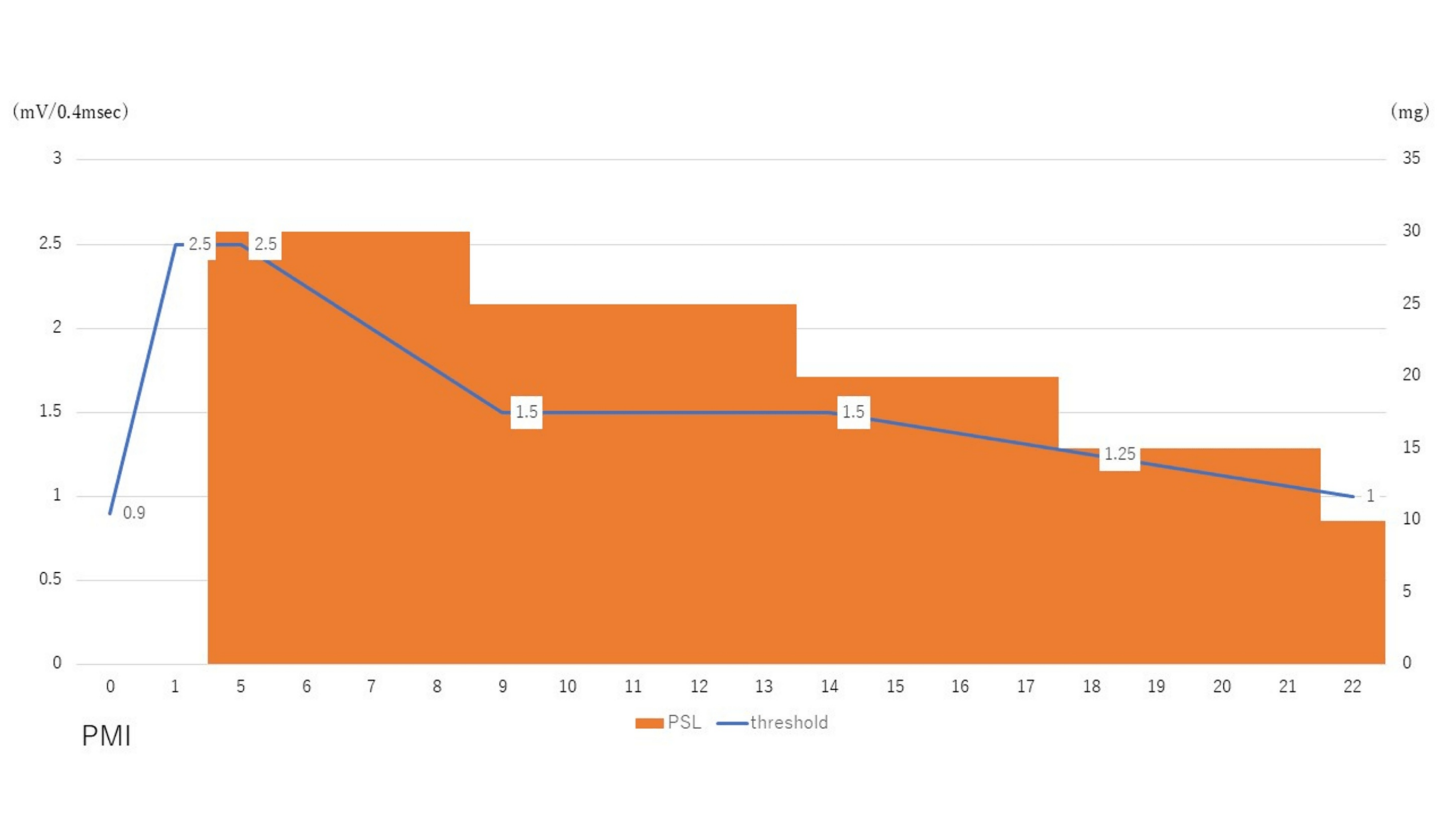
Clinical course about ventricular lead thresholds The figure shows the clinical course of the ventricular lead thresholds after pacemaker implantation. The left side of the vertical line shows the ventricular lead thresholds (mV/0.4msec), the right side of the vertical line shows the dose of prednisolone (mg), and the horizontal line shows the days after pacemaker implantation. PMI, pacemaker implantation; PSL, prednisolone

Two weeks later, the ILR revealed the frequent recurrence of syncope with occasional palpitations. ECG revealed progression to a complete AV block (Figure 3). No evidence of ventricular tachycardia was observed. A symptomatic heart block was suspected; therefore, the patient underwent permanent pacemaker implantation. A postprocedural fluorodeoxyglucose-18-positron emission tomography/computed tomography (FDG-PET/CT) was performed (Figure 4A), which showed high uptake in the lateral wall; therefore, the patient was diagnosed with isolated cardiac sarcoidosis. Incidentally, biopsy was not performed and serum markers (ACE and sIL-2R) were within normal range. After cardiac sarcoidosis was confirmed, steroid therapy was initiated using 30 mg of prednisolone. The steroid therapy was adjusted based on the Japanese Circulation Society Guidelines: 30 mg daily as prednisolone equivalent for four weeks and then the dose was tapered by 5mg daily at intervals of four weeks. Figure 5 shows the patient’s clinical course of ventricular lead thresholds after pacemaker implantation. The threshold was 0.9 mV immediately after implantation but rapidly increased to 2.5 mV one week later. Steroid therapy was effective in improving the ventricular lead threshold. Ten months later, a follow-up FDG-PET/CT showed less FDG uptake in the lateral wall compared with that observed on the initial scan (Figure 4B).

Follow-up information

The patient continues regular outpatient follow-up, with pacemaker parameters monitored remotely. To date, the patient has shown stable progress without any exacerbations. If the ventricular lead threshold gets high again, it might mean that inflammation has recurred, which would require cardiac MRI or FDG-PET/CT. Fortunately, the patient is monitored remotely, and there has been no evidence of ventricular arrhythmias; however, if ventricular arrhythmias develop, defibrillator implantation and increasing the amount of steroid would be required.

## Discussion

Herein, we reported the case of a patient with isolated cardiac sarcoidosis who was diagnosed using ILR. Upon diagnosis, a permanent pacemaker was implanted, and steroid therapy was initiated. We considered that the threshold had increased rapidly in the patient due to the spread of cardiac inflammation, which reached the tip of the lead. Early steroid therapy has proved effective in treating this condition. The long-term benefits of steroid therapy in reducing clinical morbidity and mortality in patients with cardiac sarcoidosis have been demonstrated [2]. However, the effectiveness of steroids in the treatment of cardiac sarcoidosis has not been fully elucidated [5].

A previous report showed that early steroid therapy was ineffective in approximately half of the patients with a complete AV block in whom pacemaker implantation was required [6], indicating that early steroid therapy prevented pacemaker implantation in half of the patients. Some researchers have suggested that steroid therapy should precede pacemaker implantation. However, considering the unpredictable improvement in the AV block, the possibility of recurrence, and the risk of infection prior to device implantation, the authors of the current study believe that steroid therapy should be started after pacemaker implantation. Moreover, the patient in the present case frequently had recurrent syncope and presented with palpitations. Therefore, early intervention was considered necessary. The ILR recordings were instrumental in facilitating a definitive diagnosis and initiating prompt treatment, such as administering steroid therapy.

Although the cause of syncope is difficult to determine in many cases, ILR can assist clinicians in identifying not only the cause of syncope but also the underlying disease, allowing for a relatively early intervention. When young patients with syncope are diagnosed with vasodepressor reflex syncope, additional tests are not generally performed. However, the present patient immediately experienced syncope while undergoing the head-up tilt test, and this symptom was different from the previous episodes of syncope. In addition, the patient had suspected cardiogenic syncope, a reduced EF, and a wide QRS; therefore, an ILR was considered the best approach for obtaining a differential diagnosis. An ILR is useful in patients with syncope of unknown etiology. The PICTURE study examined 570 patients without a definitive diagnosis despite undergoing an average of 13 different diagnostic tests in three different clinical departments prior to ILR implantation. Syncope recurred in 218 (36%) patients within 12 months, and the ILR led to a causative diagnosis in 170 (78%) patients [7]. In the present case, ILR contributed to diagnosing this critical disease. From the author's experience, the frequency of arrhythmias causing syncope is very rare, and thus long-term ECG monitoring such as ILR is needed, and other non-invasive methods could not reveal them. Early diagnosis is important because sarcoidosis is a progressive disease, and if the diagnosis is too late, fatal arrhythmias could occur before initiating treatment.

## Conclusions

The present report describes a patient with cardiogenic syncope, who was ultimately diagnosed with isolated cardiac sarcoidosis. An ILR was instrumental in facilitating the differential diagnosis. Steroid therapy was effective for the recovery of the lead threshold. This case confirms that an ILR is useful for patients with idiopathic syncope suspected to be cardiogenic syncope.
